# Introduction to the Biomedical Linked Annotation Hackathon (BLAH) 2015 Symposium

**DOI:** 10.1186/1753-6561-9-S5-A1

**Published:** 2015-08-06

**Authors:** Jin-Dong Kim, Kevin Bretonnel Cohen, Nigel Collier, Zhiyong Lu, Pontus Stenetorp

**Affiliations:** 1 Database Center for Life Science, Research Organization of Information and Systems, Kashiwa, Japan; 2 School of Medicine, University of Colorado, Denver, Colorado, US; 3 European Bioinformatics Institute (EMBL-EBI), Hinxton, UK; 4 National Center for Biotechnology Information (NCBI), Bethesda, MD, USA; 5 University of Tokyo, Tokyo, Japan

## 

Scientific literature is a central repository of scientific knowledge - every important scientific discovery has been published in it. As such, it has become a main target of data mining, and in particular, text mining. However, the unstructured, or covertly structured, nature of natural language texts poses a major barrier to accessing the contents of literature. The technology of literature annotation thus has played a central role for text mining. While it still requires enormous further effort, the productivity of literature annotation is recently significantly improved, and there are quite a few groups producing annotations in large scale. While many groups have released those annotation data sets openly to the public, however, the way of sharing the widely valuable resources still remains at a primitive level, e.g., relying on individual exchange of archived files.

Meanwhile, the advancement of internet web technology has enabled much convenient ways of collaborating for producing and sharing data. For example, the technology of web 2.0 has enabled crowdsourcing content generation, and web 3.0 has enabled machine-understandable web of data (WoD), a.k.a, linked data (LD), as a mean for linking and sharing data.

The first BLAH hackathon/symposium event was organized to initiate a concentrated collaboration for linking and sharing biomedical literature annotation utilizing recent web technology. Specifically, the event aimed at (1) collecting various annotations to PubMed and PMC articles, (2) linking them through normalized texts of literature, and (3) making the resources publicly available through standard Web protocol. After spending four days together during the hackathon to discuss and develop initial sets of shared resources of biomedical literature annotation, ideas for further effort were presented and discussed during a post-hackathon one-day symposium. In the proceedings, extended abstracts of five projects presented in the symposium are included. For the entire program and the output of the hackathon, readers are referred to the homepage of the event: http://2015.linkedannotation.org.

We believe the output of the hackathon and the ideas discussed during the symposium has a good potential to open a new era of text mining, enabling rich analysis of heterogeneous annotations, e.g., syntactic and semantic annotations, genomic and clinical annotations, and so on, which is not possible using single annotation sets individually.

